# Universal immunization program in India: a review of childhood vaccine updates over the last decade (2014-2024)

**DOI:** 10.1016/j.ijregi.2026.100957

**Published:** 2026-07-19

**Authors:** Tintu Varghese, Rohan Michael Ramesh, Manoj Jacob Dhinagar, Nancy Angeline Gnanaselvam, Rajeev Zachariah Kompithra, Deepak Thomas Varughese, Gagandeep Kang

**Affiliations:** 1 The Wellcome Trust Research Laboratory, Department of Gastrointestinal Sciences, Christian Medical College, Vellore, Tamil Nadu, India; 2 Rural Unit of Health and Social Affairs, Christian Medical College, Vellore, Tamil Nadu, India; 3 Department of Community Medicine, St. Johns Medical College, Bangalore, Karnataka, India; 4 Department of Child Health, Christian Medical College, Vellore, Tamil Nadu, India; 5 Department of Community Medicine, Believers Church Medical College, Thiruvalla, Kerala, India; 6 Centre of Public Health, Christian Medical College, Vellore, Tamil Nadu, India

**Keywords:** Mission Indradhanush, Digital health, New vaccines, Full Immunization Coverage (FIC), National Family Health Survey (NFHS), COVID-19

## Abstract

**Objectives:**

Immunization is a cornerstone of public health, protecting children from life-threatening, yet preventable diseases. Established as the Expanded Programme on Immunization in 1978, India’s Universal Immunization Programme is the largest in the world and has undergone major transformations over the decades. This narrative review provides an overview of key developments in childhood immunization in India during the last decade (2014-2024).

**Methods:**

A literature search of PubMed, Scopus, and Google Scholar was conducted to identify relevant English-language publications from January 2014 to December 2024.

**Results:**

The review examines four major dimensions: the introduction of new childhood vaccines under the Universal Immunization Programme; trends in vaccination coverage and persistent inequities in access; the implications of the coronavirus disease 2019 pandemic on routine immunization; and recent advances in childhood vaccination within the Indian context.

**Conclusion:**

Collectively, these findings highlight India’s substantial progress toward universal immunization, while underscoring the continuing challenges in achieving equitable, resilient, and sustainable vaccine delivery systems.

## Highlights

• Reviews key developments in childhood immunization in India (2014-2024).
• Examines new vaccine introductions under the Universal Immunization Program.
• Assesses vaccination coverage trends, inequities, and access challenges.
• Evaluates coronavirus disease 2019 impacts and recent advances in childhood vaccination.

## Introduction

Vaccination stands as one of the most pragmatic and effective public health interventions in lowering the childhood mortality and morbidity on a global scale. Globally, an estimated six million deaths from vaccine-preventable diseases (VPDs) are averted each year through immunization [1]. Following the successful eradication of smallpox in 1975, India adopted the Expanded Programme on Immunization in 1978, originally launched by the World Health Organization (WHO) in 1974 [2]. In 1985, this initiative was renamed the Universal Immunization Programme (UIP), with the primary goal of achieving full national coverage and providing vaccines free of cost to all eligible beneficiaries [2].

India’s UIP is the largest immunization program in the world, in terms of the quantity of vaccines used, number of beneficiaries reached, geographical coverage, and workforce involved. Each year, the program targets approximately 29 million pregnant women and 26.7 million infants, conducting more than 12 million immunization sessions annually [3]. The UIP currently offers free vaccinations against 12 diseases. Of these, 11 diseases are addressed on a national scale, including severe forms of childhood tuberculosis, diphtheria, pertussis, tetanus, measles, rubella, polio, rotavirus diarrhea, hepatitis B, pneumococcal pneumonia, meningitis, and pneumonia caused by *Haemophilus influenzae* b. Additionally, Japanese encephalitis is targeted in certain endemic districts at a sub-national level.

**Figure 1:**
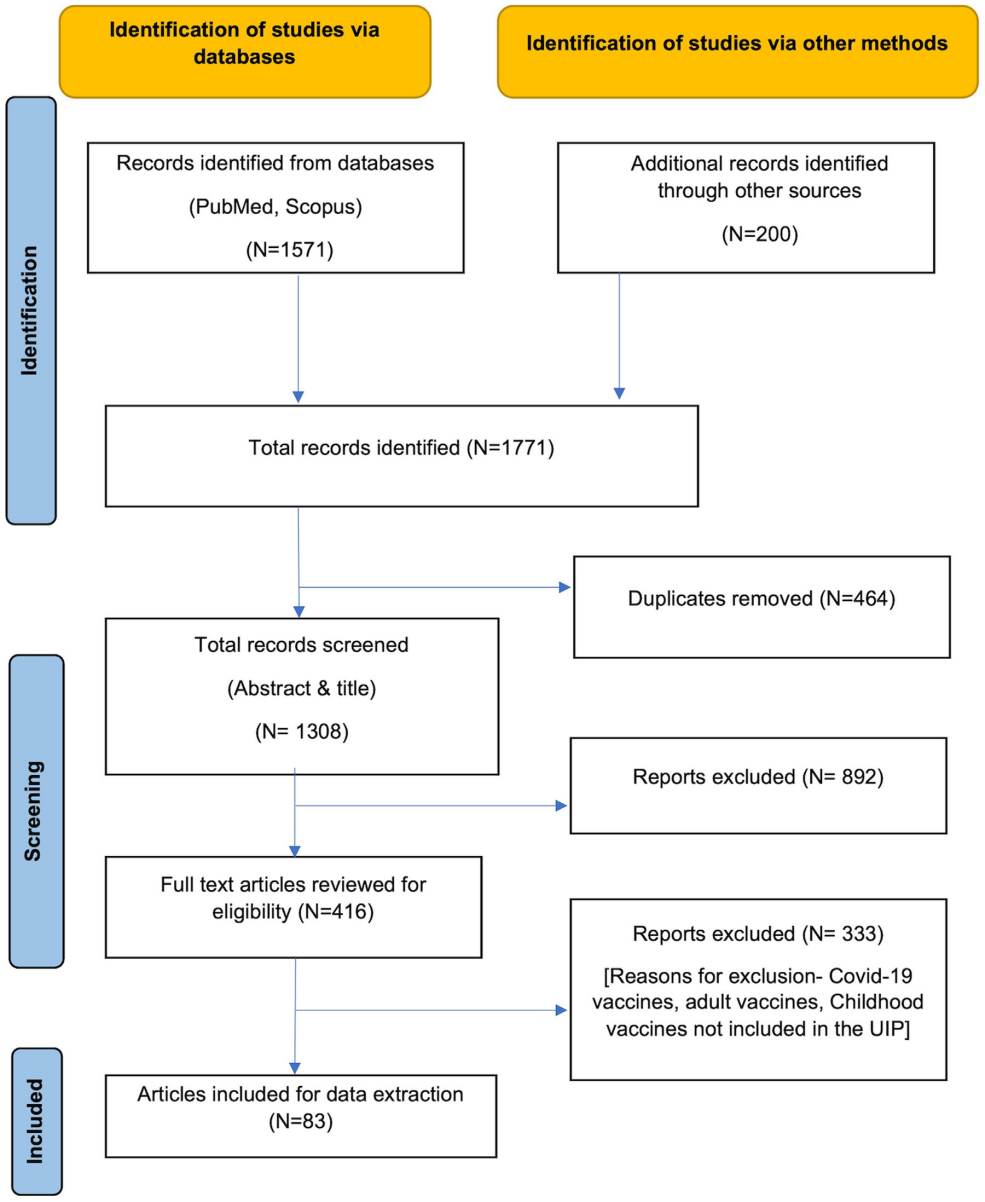
Summary of search strategy used to identify relevant literature.

Since its inception, the UIP has evolved to address the changing public health needs of the country. This narrative review aims to provide a comprehensive update on the progress and advancements in India’s childhood immunization schedule during the past decade (2014-2024). A literature search was conducted in PubMed, Scopus, and Google cholar, complemented by reports from United Nations Development Programme, United Nations Children’s Fund, and WHO. Search terms included combinations of keywords related to India, childhood vaccination, UIP, vaccine introduction, Mission Indradhanush, eVIN, and vaccine hesitancy. Articles published in English between 2014 and 2024 were included. The search yielded 1772 records, with 464 duplicates removed. Of the remaining 1308 articles, 416 met the inclusion criteria (Figure 1). Screening and data extraction were performed independently by two reviewers, and disagreements were resolved in consultation with two additional authors using the systematic review web application, Rayyan (https://rayyan.ai). This narrative review examines four key dimensions of India’s UIP [4]:

1. New childhood vaccines introduced under the UIP in the last decade
2. Current vaccination coverage rates and persistent disparities in vaccine access
3. The implications of the coronavirus disease 2019 (COVID-19) pandemic on immunization services and lessons learned
4. Recent scientific advances in childhood vaccination within the Indian context

## Evolving landscape of childhood vaccination in India (2014-2024)

**Figure 2:**
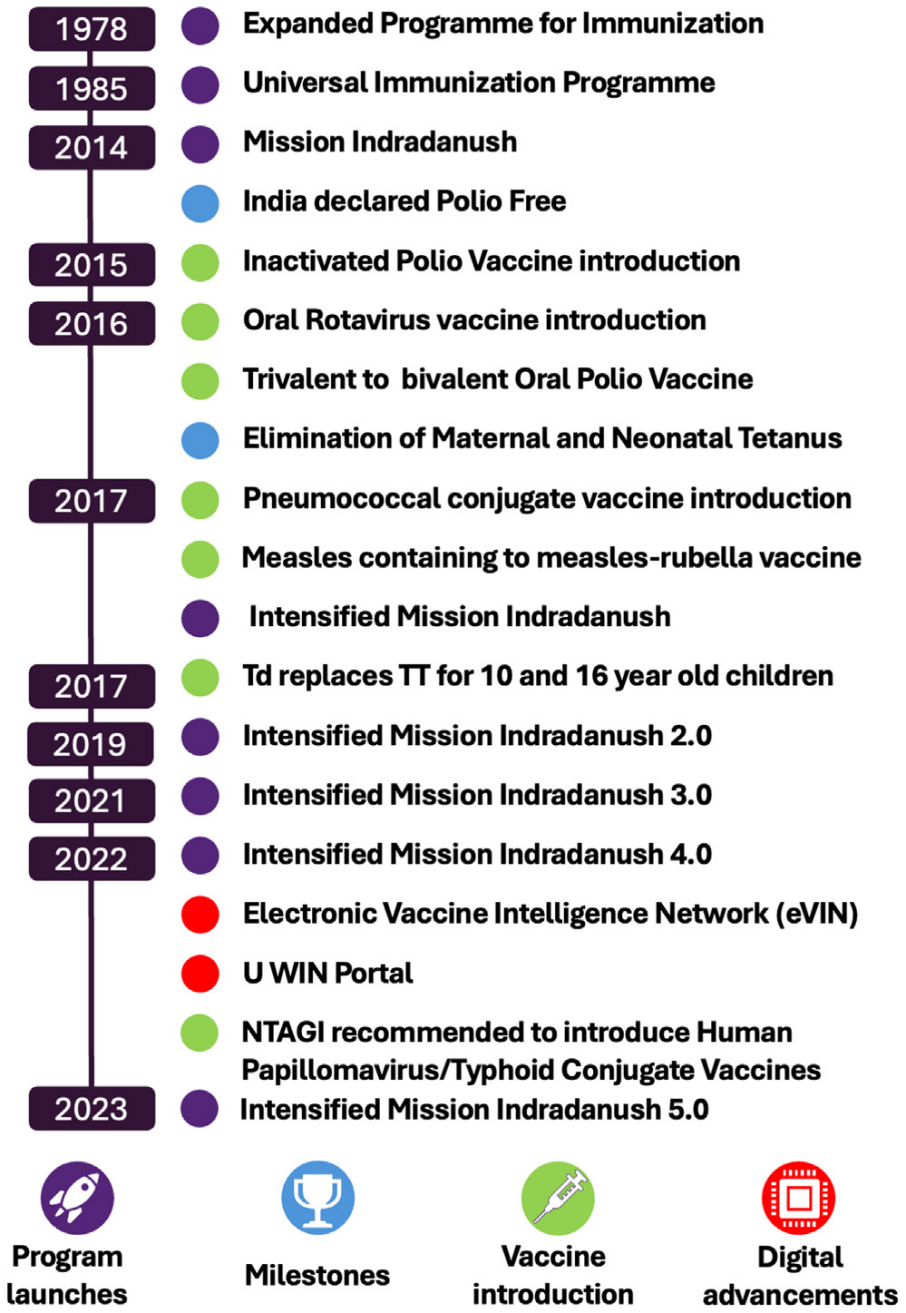
Key milestones and changes in Indian UIP from 2014 to 2024. UIP, Universal Immunization Program.

Over the past decade, India’s UIP has undergone rapid expansion and transformation, characterized by the introduction of new vaccines, launch of Mission Indradhanush to enhance equity and outreach, strengthened surveillance of VPDs, and adoption of digital platforms for real-time monitoring. These collective reforms have driven immunization coverage to unprecedented levels and yielded landmark achievements such as polio eradication (March 2014), elimination of maternal and neonatal tetanus (July 2016), and significant progress toward measles-rubella elimination [3]. Figure 2 outlines the key milestones and transitions in the UIP during this period, which are discussed in detail in the following sections.

## Expanding protection: new vaccines in the UIP

The childhood immunization program in India, initially comprising Bacille Calmette-Guérin (BCG), Oral Polio Vaccine (OPV), and Diphtheria, Pertussis, and Tetanus (DPT) vaccines, was progressively expanded with the addition of the measles vaccine in 1985 and Hepatitis B (HepB) as the seventh antigen in 2011 [3,4]. The last decade (2014-2024) has witnessed substantial expansion of the UIP, marked by the nationalization of *Haemophilus influenzae* type b (Hib) as part of the pentavalent combination vaccine (DPT-HepB-Hib); introduction, scale-up and nationalization of Inactivated Poliovirus Vaccine (IPV), Measles-Rubella (MR) vaccine, Oral Rotavirus Vaccine (ORV), Pneumococcal Conjugate Vaccine (PCV), and Tetanus; and reduced concentration of Diphtheria vaccine (Td), thereby significantly broadening protection against VPDs.

The introduction of new vaccines under the UIP is informed by rigorous technical evaluation and programmatic feasibility assessments. The National Technical Advisory Group on Immunization (NTAGI) evaluates disease burden (incidence, prevalence, morbidity, mortality, and epidemic potential), vaccine safety and efficacy, cost-effectiveness compared with alternative interventions, and alignment with existing vaccination schedules [5]. The Mission Steering Group, the highest policy-making body under the National Health Mission, provides broad policy direction by assessing programmatic feasibility, including vaccine production capacity, financial sustainability, and implementation readiness and oversees overall program governance for the health sector [6].

India employs three primary strategies for new vaccine introduction under the UIP:

1. Campaign followed by routine introduction—used when rapid coverage is essential due to technical or public health reasons (e.g., MR vaccine)
2. Routine introduction—integrating new vaccines into existing schedules with a defined cutoff date, often linked to current vaccines (e.g., ORV, PCV, and IPV introduced alongside the first pentavalent dose)
3. Replacement strategy—phased substitution of existing vaccines with newer formulations once prior stocks are utilized (e.g., trivalent OPV [tOPV] to bivalent OPV [bOPV], Measles to MR)

In the past decade, these strategies have enabled the successful introduction of PCV, ORV, IPV, and Pentavalent vaccine. Concurrently, several vaccine type transitions have occurred—such as tOPV to bOPV, DPT + HepB to Pentavalent, Measles to MR, and Tetanus Toxoid to Td—alongside schedule revisions, including shifting Measles and Japanese Encephalitis vaccines from single to two-dose regimens and modifying IPV from a two-dose to a three-dose fractional schedule. The UIP has also adopted vaccine product interchangeability, accommodating different formulations of ORV, Japanese Encephalitis, PCV, and MR vaccines, enhancing program flexibility and sustainability.

## Pentavalent vaccine: inclusion of *Haemophilus influenzae* type b

In 2008, the NTAGI recommended national rollout of Hib vaccine as pentavalent vaccine [7]. This was based on estimated significant disease burden in India (approximately 2.4 million Hib cases and 72,000 deaths annually in children under five years, accounting for 4% of overall childhood mortality) alongside global safety and efficacy data of pentavalent vaccines used elsewhere [8,9].

The pentavalent vaccine was initially piloted in Tamil Nadu and Kerala in 2011, later expanding to six additional states by early 2013 [7]. A nationwide scale-up followed in two phases—first in 12 states by late 2014 and then across the remaining 16 states by mid-2015, achieving full national coverage within four years [7,10]. Although Hib vaccine had been available in India's private sector for nearly a decade, its introduction into the UIP ensured equitable access, particularly for the poorest children at highest risk.

Initial challenges included low Hib disease awareness among healthcare providers and the public, leading to slow vaccine uptake and demand. Limited surveillance capacity made accurate disease burden measurement and vaccine impact monitoring difficult in early implementation years [10]. A few adverse events and deaths temporally associated with the vaccine were highlighted in the media; however, these could not be causally linked to vaccination, and subsequent studies confirmed vaccine safety and efficacy [11]. As a combination vaccine, the pentavalent vaccine offers multiple advantages such as multiple antigens in a single shot, reducing injections, production of hazardous sharps waste and exposure to preservatives/stabilizers. Programmatically, it streamlines logistics, saves cold chain space, lowers costs, and enhances immunization coverage and schedule completion [12].

## Switch to bivalent oral polio vaccine, introduction and scale-up of inactivated poliovirus vaccine

Following the global eradication of wild poliovirus type 2, the Global Polio Eradication Endgame and Strategic Plan (2013-2018) mandated a coordinated global transition from the trivalent to the bivalent (which covers only types 1 and 3) OPV in April 2016 [[13], [14], [15]]. The Sabin poliovirus type 2 was removed from the tOPV and this switch was designed to minimize the risk of circulating vaccine-derived poliovirus type 2, enhancing safety while ensuring continued protection against the remaining poliovirus types. A central focus of the withdrawal process was toward systematically identifying and eliminating the remaining tOPV vaccine stocks/supplies [16].

Before the transition, the Strategic Advisory Group of Experts on Immunization, WHO, recommended countries to introduce at least one dose of IPV in their routine immunization schedules [14]. IPV, a killed virus vaccine, boosts immunity without risk of vaccine-derived virus shedding, thereby maintaining population immunity against type 2 poliovirus. Combined use of bivalent OPV and IPV sustained herd immunity and prevented both wild and vaccine-derived poliovirus outbreaks. Aligned with global recommendations, the Government of India introduced full-dose IPV in a phased manner across six states in November 2015, primarily Global Alliance for Vaccines and Immunisation (Gavi)-funded but substantially supplemented by domestic resources [14].

However, WHO announced a global IPV shortage in February 2016, projected to persist until 2020. Amid anticipated IPV shortages and strong evidence that two fractional intradermal IPV doses provide equal or superior immunogenicity to a single full intramuscular dose, India adopted a routine fIPV schedule as a risk-mitigation strategy [17]. India became the first country to incorporate fIPV into its routine immunization program in a phased manner, starting with eight states in 2016 [16]. fIPV is administered as a two-dose schedule of 0.1 mL intradermally at 6 and 14 weeks of age, replacing a single intramuscular full dose of 0.5 mL. Fractional dosing uses one-fifth (0.1 mL) of the full intramuscular dose (0.5 mL), enabling vaccination of more children with the same vaccine quantity, reducing costs, and easing supply constraints. The transition also created additional training and supervision needs, along with challenges in dose-tracking, forecasting, and record-keeping, especially during the period when full-dose and fractional-dose vaccine were used in parallel. Wastage was another operational concern, particularly in sparsely populated areas where smaller vial sizes were needed to reduce losses and maintain efficiency [17]. After a swift assessment of the implementation, fractional dosing was expanded to the entire country by June 2017, including the transition of states that initially used the full-dose IPV [16]. Training, accurate vaccine demand forecasting, wastage minimization, meticulous record-keeping, and close supervision ensured smooth transition from full to fractional dosing. In January 2023, based on evidence demonstrating equivalent seroconversion rates between three doses of fIPV and three full doses of IPV, a third fIPV dose was added to the UIP schedule, administered between 9 and 12 months of age [17].

## Oral rotavirus vaccines

The introduction of the rotavirus vaccine in India represents a unique and landmark public health achievement, resulting from decades of collaborative research since the discovery of an unusual rotavirus strain in 1985 [18]. India introduced two indigenously developed rotavirus vaccines—Rotavac and Rotasiil—into its UIP, becoming the first country in the WHO South-East Asia Region to do so. Rotavac, developed by Bharat Biotech, is derived from the naturally attenuated bovine-human reassortant 116E strain (G9P[11]), whereas Rotasiil, produced by the Serum Institute of India, is a pentavalent bovine-human reassortant vaccine (G1-G4, P [8]) [19].

Following the NTAGI’s recommendation, rotavirus vaccines were introduced in phases, starting in four states and subsequently expanded to nationwide coverage, from March 2016 to April 2020 [20]. The decision to introduce rotavirus vaccines was based on robust national evidence from a multisite surveillance network, which revealed that rotavirus accounted for approximately 40% of gastroenteritis-related hospitalizations among children under five [21]. With India accounting for 22% of global rotavirus-related deaths and an annual birth cohort of 26 million children, the public health rationale for vaccine introduction was thus compelling [22]. Post-introduction surveillance indicates a marked reduction in rotavirus-associated hospitalizations, with test positivity declining from 40% pre-vaccine to around 20%, demonstrating population-level impact despite moderate efficacy of 54% (95% CI 35.0-66.9) [23].

Both Indian vaccines are priced at under $1 per dose for public programs—compared with ∼$15 per dose for Rotarix and Rotateq, making them among the world’s most affordable rotavirus vaccines [24]. With WHO prequalification, both vaccines became eligible for global procurement through United Nations Children's Fund and Gavi, and are now increasingly used in other low- and middle-income countries as multinational supplies decline. Ongoing studies are exploring strategies to enhance vaccine performance in Indian settings, including neonatal dosing, booster doses at 9 months, and injectable rotavirus vaccines, to further optimize protection and sustain gains in child health [23].

## Pneumococcal conjugate vaccine

Pneumococcal disease continues to be one of the foremost vaccine-preventable causes of mortality in children under five years of age worldwide and in India. India bears nearly one-fourth of the global childhood pneumonia burden, with an estimated 8 million severe cases annually, over half requiring hospitalization or intensive care and pneumococcal infections accounting for at least 0.1 million under-five deaths every year [25]. In 2015, the NTAGI recommended the introduction of the pneumococcal conjugate vaccine (PCV) into the UIP, selecting PCV13 (Prevnar), a WHO-prequalified vaccine compatible with the open vial policy, as the preferred option [26]. With Gavi support, phase 1 introduction (2017-2020) began in five high-burden states, following a three-dose schedule at 6 weeks, 14 weeks, and 9 months [27,28]. Despite COVID-19-related challenges, the national rollout (2020-2021) successfully extended PCV coverage to all remaining states and union territories [27].

In 2021, PCV10 (Pneumosil), the first indigenously developed pneumococcal vaccine, became available, ensuring uninterrupted domestic supply during the pandemic [29]. By 2024, a second locally produced vaccine, PCV14 (Pneubevax 14), was approved, expanding protection against additional pneumococcal serotypes and gradually replacing PCV13 in the UIP [30]. Together, PCV10 and PCV14 provide broad coverage against the major disease-causing strains of *Streptococcus pneumoniae*. Since the introduction of the vaccine, a substantial decline in pneumonia-related under-five mortality has been reported however, this evidence is preliminary and should be interpreted in the context of concurrent child health interventions rather than attributed to PCV alone [27]. Post-PCV surveillance should monitor serotype replacement, emerging non-vaccine serotypes, and antimicrobial resistance, because Indian data show persistence of drug-resistant serotypes 19F and 19A and an increase in non-vaccine serotype 15B. Such surveillance is essential to guide future vaccine-valency decisions and antimicrobial resistance control strategies [31].

## Measles-Rubella vaccine

In 2011, the UIP added a second dose of the measles containing vaccine (MCV2, given at 16-24 months), supplementing the single-dose schedule (MCV1 at 9 months) that had been in place since 1985. In 2017, India introduced the combined measles-rubella (MR) vaccine in line with the South-East Asia Measles and Rubella Elimination Plan [32]. To boost coverage, a nationwide MR campaign (2017-2020) vaccinated all children aged 9 months to under 15 years, regardless of prior vaccination or disease history. Following the campaign, India implemented a two-dose MR vaccine strategy in routine immunization, replacing MCV1 and MCV2 [33].

Recognizing the urgency of eliminating measles and rubella, India launched the *Roadmap to Measles and Rubella Elimination in India by 2023* in September 2022 [34]. The roadmap integrates high two-dose MR coverage (targeting >95%) with strengthened surveillance, outbreak control, community engagement, and periodic assessments to identify vulnerable populations. This emphasized rapid, contiguous campaign coverage to build herd immunity, avoiding prolonged or phased rollouts that could leave pockets of unprotected children. By 2021, India transitioned from outbreak-based to case-based acute fever and rash surveillance nationwide [34]. As of 2024-25, MR vaccination coverage has reached 93.7% for the first dose and 92.2% for the second, with the country now aiming to achieve measles and rubella elimination by 2026 [35]. Despite substantial coverage gains, India did not achieve the 2023 measles-rubella elimination target, largely because zero-dose clusters and subnational coverage gaps persisted, sustaining outbreak risk in several states and districts [36].

## Reaching every child: lessons from Mission Indradhanush

Launched in December 2014, Mission Indradhanush (MI) aimed to accelerate India’s progress toward universal immunization by reaching unvaccinated and under-vaccinated children through targeted campaigns. The goal was to achieve 90% full immunization coverage (FIC) by 2020, up from 65% in 2014, by reallocating resources to underserved regions and conducting four special rounds of immunization each year [36,37]. FIC is defined as the proportion of all children aged 12-23 months receiving one dose each of BCG and measles vaccines, along with three doses each of DPT and OPV [38]. Building on the success of the Pulse Polio Program, MI prioritized high-risk and hard-to-reach populations—such as those in urban slums, tribal and hilly areas, and migratory communities. The mission also addressed systemic gaps like vacant health subcenters, outbreaks, dropout rates, vaccine hesitancy, and workforce shortages. Following its initial two phases, MI led to a 6.7% overall rise in full immunization coverage (7.9% in rural and 3.1% in urban areas) [39].

Despite early gains, urban areas continued to show lagging progress, prompting the launch of the Intensified Mission Indradhanush (IMI) in 2017 [39]. IMI focused on districts and cities with persistently low coverage, introducing intersectoral collaboration beyond the health sector. IMI improved complete immunization coverage in targeted areas to 69%—an 18.5% increase from pre-IMI levels, reaching nearly one-third of children who would otherwise have missed vaccination [40].

Subsequent phases—IMI 2.0 (2019), IMI 3.0 (2021), IMI 4.0 (2022), and IMI 5.0 (2023) focused on recovering missed doses during the COVID-19 pandemic, addressing high-risk districts, and extending coverage to children up to five years of age, with special emphasis on measles-rubella elimination [41]. Collectively, MI and IMI campaigns vaccinated approximately 6 million children and 0.5 million pregnant women per round, contributing to a 14.4% increase in FIC—from 62% (NFHS-4, 2015-16) to 76.4% (NFHS-5, 2019-21) [42].

According to WHO’s VPD surveillance data, from 2014 to 2021, these initiatives prevented an estimated 321,847 vaccine-preventable disease cases and 4002 deaths [43]. Beyond coverage gains, MI and IMI strengthened India’s immunization system through evidence-based microplanning, intersectoral coordination, community engagement, improved cold-chain logistics, data quality, and adverse event monitoring. A key lesson from this initiative is the emphasis on equity and inclusivity, ensuring that children in underserved and high-risk populations are not left behind in India’s immunization journey.

## Unequal gains: understanding coverage gaps and access disparities

Between National Family Health Survey (NFHS)-4 (2015-16) and NFHS-5 (2019-21), India’s UIP achieved substantial gains. FIC increased from 62.0% to 76.6%, whereas the proportion of completely unvaccinated children (zero-dose) declined from 6.0% to 3.6% [44,45]. Coverage improved across all major vaccines. BCG maintained the highest uptake, rising from 91.9% to 95.2%. The DPT series recorded notable gains—first-dose coverage increased from 89.5% to 93.6%, second-dose from 85.7% to 91.4%, and third-dose from 78.4% to 86.9%—reducing dropout from 11.1% to 7.2%. OPV showed modest improvements, with coverage for dose-1, dose-2, and dose-3 increasing from 90.8%, 86.0%, and 72.8% to 92.4%, 88.5%, and 80.5%, respectively, though dropout remained relatively high at 12.9%. Hepatitis B vaccination registered the largest relative improvement, with third-dose coverage climbing from 62.7% to 87.0% (Penta) [44,45]. State-specific interactive maps illustrating changes in vaccine coverage between NFHS-4 and NFHS-5 for each vaccine are available through this [46].

New vaccines introduced between surveys: ORV coverage had reached 44.0% (dose 1)**,** 36.4% (dose 2)**,** and 35.3% (dose 3), with a 19.8% dropout between the first and third doses—reflecting typical early-phase challenges of newly introduced vaccines [47]. According to the 2024 WUENIC (WHO and UNICEF Estimates of National Immunization Coverage) estimates, dose-3 rotavirus vaccine coverage has improved, reaching 92%, indicating continued progress [47]. Although NFHS-5 did not fully capture PCV coverage, operational data indicate booster uptake rose from 25% in 2021 to 83% by 2023, demonstrating successful expansion beyond the survey period [27,48].

## Regional disparities persisted despite overall improvement

**Figure 3:**
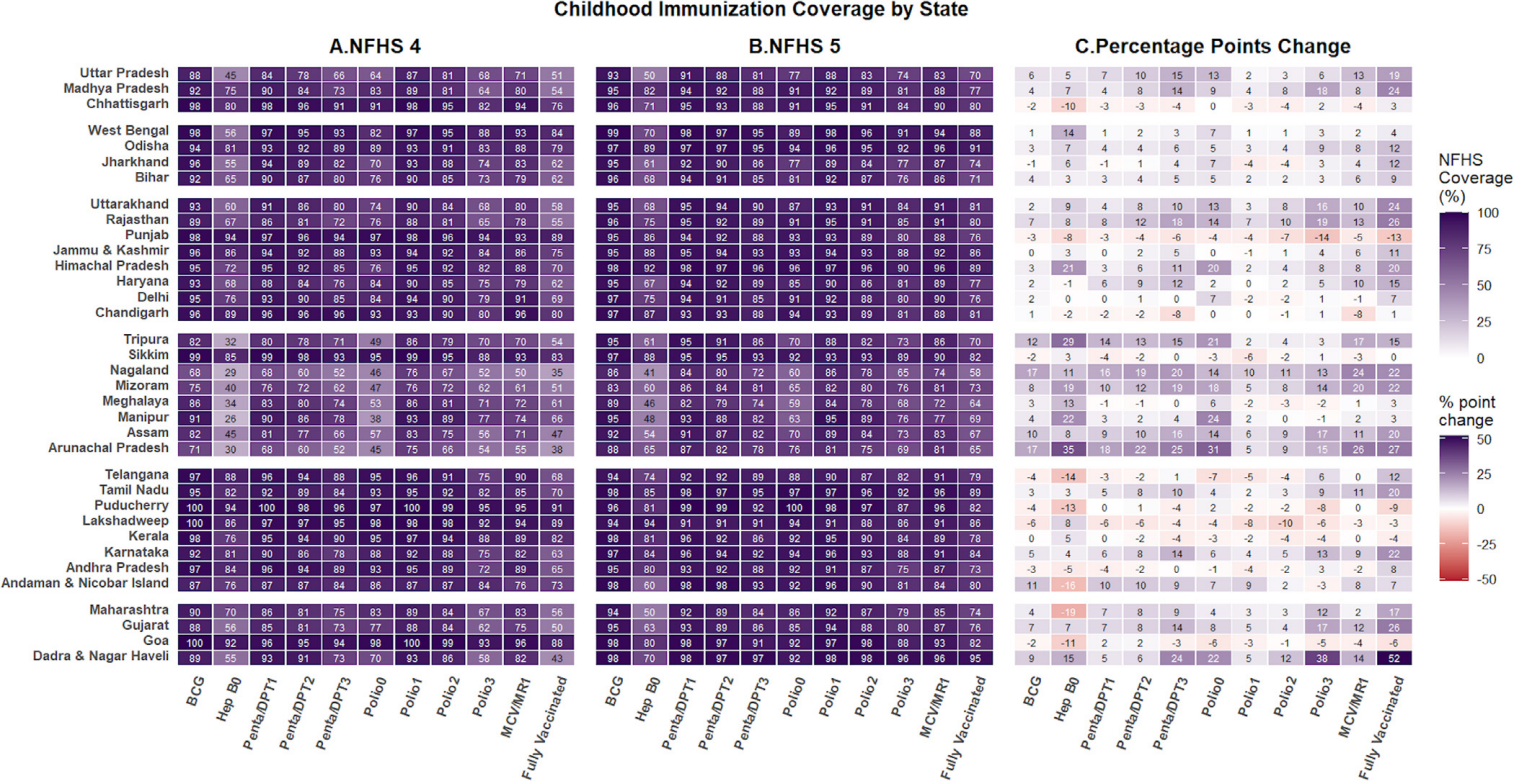
Childhood immunization coverage by state/union territory in infants.

Despite overall improvements, significant regional and intra-state disparities persist. States such as Dadra & Nagar Haveli/Daman & Diu (95%), Odisha (91%), and Tamil Nadu (89%) achieved high FIC, while several northeastern states—including Nagaland (58%), Meghalaya (64%), and Arunachal Pradesh (65%) remained below 70% [49,50]. These geographically challenging regions had lower overall immunization coverage (68.4%), higher dropout rates (25%), and greater proportion of unvaccinated children (7.0%). District-level analysis further revealed stark intra-state inequities: among 707 districts, 50 reported FIC below 50%, clustering in the northwestern (Rajasthan, Gujarat, parts of Madhya Pradesh and Maharashtra), northeastern (Arunachal Pradesh, Assam, Nagaland), and central (notably Madhya Pradesh) regions. Even relatively high-performing states such as Tamil Nadu and Karnataka contained districts with suboptimal coverage (<80%), underscoring persistent inequities in vaccine access and delivery [51]. Overall, subnational heterogeneity persists across zones, with some union territories and western/northern states approaching near-universal coverage for multiple doses, while parts of northeast and central India continue to trail especially on third-dose completion and MR. The coverage of the birth dose of Hepatitis B and OPV consistently underperform in all zones. Taken together, the pattern suggests that the largest marginal gains have accrued where baseline coverage was modest and multi-antigen improvements were feasible, whereas jurisdictions with historically high coverage exhibit smaller net changes or declines, indicating the need for targeted strategies to reduce drop-out, sustain high performance, and close remaining equity gaps (Figure 3).

## Other reasons for vaccine access inequity

Persistent inequities in vaccine access are driven by structural and social determinants such as gender norms limiting women’s mobility, marginalization of tribal and lower-caste communities, poor transport connectivity, urban slum exclusion, and economic precarity among migrant workers. NFHS-4 and NFHS-5 show strong social gradients in immunization, with lower coverage among children of mothers with limited schooling and in the poorest wealth quintiles, and intersecting effects of religion and gender in some states [52]. Discrimination, digital divides, and conflict-driven displacement further hinder equitable uptake, revealing that universal coverage remains constrained by social and geographic inequities, not vaccine availability [53,54].

Another important factor is vaccine hesitancy (reluctance or refusal to vaccinate despite the availability of services). WHO has identified vaccine hesitancy as one of the top ten threats to global health, and it is increasingly recognized as a constraint on India’s progress toward universal immunization [55]. Emerging evidence from Indian settings links parental concerns about adverse events, misinformation (including through social media), religious and cultural beliefs, and mistrust of institutions with missed or delayed doses, contributing to persistent zero-dose clusters and subnational coverage gaps, particularly in some tribal, urban-slum, and migrant communities [56,57].

## Routine childhood vaccination during the COVID-19 pandemic

Routine immunization services in India experienced significant setbacks during the COVID-19 pandemic. National DPT3 coverage dropped to 85% in both 2020 and 2021, with individual vaccine coverage declining by 2-10% and timely vaccination rates falling by 3-5% through April 2021 [58]. Multiple barriers contributed to these declines, including parental and health worker concerns about infection risk, transportation restrictions during lockdowns, economic hardship, supply chain disruptions, and diversion of health resources toward pandemic response. The most severe disruptions occurred in April 2020, particularly in districts with stringent lockdowns and limited health system capacity, followed by a second decline during the 2021 surge [59].

Despite these challenges, the pandemic accelerated key innovations within India’s immunization program. Digital tools such as eVIN and U-WIN enhanced real-time vaccine stock management, monitoring, and scheduling, while increased public and policy attention to vaccination strengthened infrastructure, outreach, and system resilience [60]. By 2023-24, immunization coverage had not only recovered but exceeded pre-pandemic levels, reflecting the system’s adaptability and growing capacity for sustained routine immunization delivery [55].

## Advancing childhood vaccination through digital solutions

Digital health innovations have transformed India’s UIP, addressing long-standing challenges in coverage tracking, service delivery, and supply chain management.

**National Electronic Immunization Registries:** Building on the success of the Co-WIN platform, the U-WIN system—launched nationwide in 2024—became the world’s largest electronic immunization registry [61]. Linked to Ayushman Bharat Health Account numbers IDs, it enables digital registration and tracking of every pregnant woman and child, provides automated SMS reminders, QR-coded vaccination certificates, and supports offline functionality. By the end of 2024, U-WIN had registered over 74.3 million beneficiaries, recorded 277.7 million vaccine doses, and facilitated 12.6 million vaccination sessions nationwide, laying the groundwork for fully digital, equity-driven immunization tracking in India [56].

**Electronic Vaccine Intelligence Network (eVIN):** Launched in 2015, eVIN is a digital system developed by India’s Ministry of Health and Family Welfare in partnership with United Nations Development Programme, supported by Gavi [62]. It modernized India’s vaccine supply chain by replacing manual processes with real-time monitoring and cloud-based data management. Digital temperature sensors placed in cold chain equipment record storage conditions every ten minutes, with hourly uploads to a central server and automatic SMS alerts to staff if temperatures go beyond safe limits or equipment fails. This system has greatly improved vaccine availability and reduced stock-outs by enabling daily digital stock updates and real-time visibility of supplies through online dashboards [62].

**Advanced Data Architecture:** A major challenge in India’s immunization ecosystem is the fragmentation of data systems, as many digital innovations have developed independently. In this context, blockchain-based architectures have been explored only in pilot settings as one potential approach to improving data integrity and traceability across systems such as the Mother and Child Tracking System and eVIN [63,64]. The Anveshan initiative, co-funded by the Bill & Melinda Gates Foundation and the Biotechnology Industry Research Assistance Council, a public sector enterprise under the Department of Biotechnology, Government of India, is a limited-scale proof-of-concept that uses tamper-proof, time-stamped records to enable secure data sharing between facilities, but remains experimental and is not yet integrated into routine UIP operations [65].

**Mobile Health (mHealth) Interventions:** mobile phone-based reminders and tracking improve immunization coverage. SMS and voice reminders have proven effective even in low-literacy communities, though impact varies by geography and population group, particularly among women and lower-income households with limited phone access or poor network coverage [66]. Computerized due-lists and global positioning system-based mapping tools like REACH help identify missed children and improve microplanning. Digital tools have also strengthened workforce capacity by providing interactive, efficient, and scalable training to health workers while improving data quality [67].

**Emerging Technologies:** Drone-based vaccine delivery has been successfully piloted in parts of northeast India, particularly in hilly areas with poor road connectivity [68]. These drones can carry up to 4.5 kg of vaccines over 17 km within about 30 minutes, making them suitable for remote delivery of routine childhood vaccines. Drone-based vaccine delivery, piloted in northeast India to overcome terrain barriers, improved accessibility in hard-to-reach areas [69]. Iceless vaccine carriers, using temperature-regulated technology to prevent freezing or overheating, further enhanced last-mile delivery efficiency [70].

Finally, digital dashboards have become essential tools for program monitoring. Data from public health facilities are integrated into the Health Management Information System, allowing real-time visualization of coverage rates, vaccine-specific uptake, dropout trends, and utilization patterns. The Immunization Control Room, within the Immunization Technical Support Unit, uses automated tracking and analytics to improve microplanning, monitor vaccine distribution, and minimize wastage—enabling data-driven decision-making at national and local levels [71].

However, these digital innovations face important limitations: digital divide and connectivity gaps, fragmented data systems, and varying data quality can hinder their effectiveness, particularly in rural and underserved settings [72]. Frontline workers also require ongoing training and support to manage new digital workflows, and there is a risk that populations with limited phone or internet access such as the urban poor and migrant families may be missed if equity is not explicitly built into system design and monitoring [73]. Recognizing and addressing these constraints will be essential to ensure that digital health tools strengthen, rather than widen, immunization gaps.

## Emerging research and future directions for India’s UIP

India’s UIP is entering a new phase of expansion following the NTAGI’s 2022 recommendation to introduce two new vaccines—the Typhoid Conjugate Vaccine (TCV) and the Human Papillomavirus (HPV) vaccine [74].

With India accounting for nearly one-third of global typhoid cases and high disease incidence confirmed by the Surveillance of Enteric Fever in India study, the inclusion of TCV represents a major public health opportunity [75]. Three WHO-prequalified TCVs—Typbar TCV®, TYPHIBEV®, and ZyVac®—are already licensed domestically, and India’s first public-sector TCV rollout in Navi Mumbai has provided encouraging safety and effectiveness evidence [76,77]. Integrating TCV into the UIP, particularly targeting urban and school-aged children, will require operational research, sustainable financing, and continued antimicrobial resistance monitoring to ensure equitable nationwide implementation.

India is also preparing for a landmark HPV vaccine rollout to advance cervical cancer prevention. The indigenously developed quadrivalent HPV vaccine (Cervavac®), approved in 2023, offers an affordable solution targeting HPV types 6, 11, 16, and 18. NTAGI recommends its inclusion for girls aged 9-14 years through school-based delivery platforms, in line with WHO’s global cervical cancer elimination strategy [78,79]. Pilot programs in Sikkim and Bihar demonstrate early success, but national scale-up will hinge on overcoming supply and financing gaps and addressing residual public hesitancy through sustained awareness campaigns and stakeholder engagement [80].

Looking forward, India is also advancing vaccine innovation for vector-borne diseases, with the indigenous tetravalent dengue vaccine DengiAll® in Phase 3 trials and local production of Takeda’s QDENGA® expected by 2026 [81]. The R21/Matrix-M™ malaria vaccine, co-developed by Oxford and the Serum Institute of India, received WHO prequalification in 2023, alongside India’s first indigenous recombinant malaria vaccine, AdFalciVax®, developed by Indian Council of Medical Research [82,83]. These developments position India not only to strengthen domestic immunization coverage but also to drive global vaccine access, innovation, and self-reliance through evidence-based policymaking and strategic investment in public health infrastructure.

## Strategic priorities for UIP toward 2030

India’s UIP aims to achieve over 95% vaccination coverage by 2030, in line with national and global goals. The focus is on reaching every child, especially those who have never been vaccinated, and ensuring vaccines are available to all. The U-WIN digital platform can track every child’s vaccination in real time and link with birth and health records for better planning and follow-up. Combination hexavalent vaccines containing IPV (Diphtheria–Tetanus–Pertussis Vaccine-HepB-Hib-IPV) are now available in India’s private sector and would be beneficial if introduced into the UIP, as it offers fewer injections, simplified schedules, and improved compliance [84]. By 2030, new vaccines such as HPV, TCV, dengue, and malaria will be considered for introduction in UIP based on expert recommendations and disease burden. Strong political support, digital tools, and a well-trained health workforce will help India sustain progress and make immunization more equitable and accessible for every child.

## Strengths and limitations

This review offers a comprehensive synthesis of a decade of childhood immunization developments in India, with a multidisciplinary scope that links policy changes, coverage trends, pandemic impacts, and scientific advances, providing relevant insights for researchers and immunization program stakeholders. However, the review is limited by its reliance on English-language publications and selected databases, which may have resulted in the omission of regional or gray literature. As a narrative synthesis, the findings are descriptive rather than quantitative, and variations in study quality and data reporting, particularly during the COVID-19 period, could not be fully accounted for. Additionally, program developments occurring after December 2024 may not be reflected in the review.

## CRediT authorship contribution statement

**Tintu Varghese:** Writing – review & editing, Writing – original draft, Software, Project administration, Methodology, Formal analysis, Data curation, Conceptualization. **Rohan Michael Ramesh:** Writing – review & editing, Writing – original draft, Visualization, Methodology, Formal analysis, Data curation, Conceptualization. **Manoj Jacob Dhinagar:** Writing – review & editing, Writing – original draft, Methodology, Formal analysis. **Nancy Angeline Gnanaselvam:** Writing – review & editing, Writing – original draft, Methodology, Formal analysis, Data curation. **Rajeev Zachariah Kompithra:** Writing – review & editing, Writing – original draft, Methodology. **Deepak Thomas Varughese:** Writing – original draft, Visualization, Methodology. **Gagandeep Kang:** Writing – review & editing.

## Declaration of competing interest

The authors have no competing interests to declare.

## Appendix Supplementary materials

Image, application 1
