# Supplementary material for: Universal immunization program in India: a review of childhood vaccine updates over the last decade (2014-2024)
Source: IJID Reg. 2026 Jul 19;20:100957. doi: 10.1016/j.ijregi.2026.100957 (PMC13486357; doi:10.1016/j.ijregi.2026.100957)
Supplement: Supplementary file 1 [file mmc1.docx]

[50]	Mathew JL. Inequity in childhood immunization in India: a systematic review. Indian	Pediatr. 2012 Mar;49(3):203–23.

[51]	Tiwari A, Sadhu G. Reimagining Public Health: Unravelling Challenges to Routine Immunization Access in Urban Communities for Migrants, the Urban Poor, and Underserved Groups. Journal of the Epidemiology Foundation of India [Internet]. 2024 June 18 [cited 2025 Nov 6];2(2):42–5. Available from: https://efi.org.in/journal/index.php/JEFI/article/view/22

[52]	Chakrabarti A, Bair EF, Thirumurthy H. Routine child immunizations in India during the COVID-19 pandemic. SSM - Population Health [Internet]. 2023 June 1 [cited 2025 Nov 6];22:101383. Available from: https://www.sciencedirect.com/science/article/pii/S2352827323000484

[53]	Sharma M, Singh SK, Sharma L, Dwiwedi MK, Agarwal D, Gupta GK, et al. Magnitude and causes of routine immunization disruptions during COVID-19 pandemic in developing countries. J Family Med Prim Care [Internet]. 2021 Nov [cited 2025 Nov 6];10(11):3991–7. Available from: https://pmc.ncbi.nlm.nih.gov/articles/PMC8797101/

[54]	Kumar P, Chakraborty AB, Dhandore S, Dhalaria P, Singh AK, Agarwal D, et al. Balancing Routine and Pandemic: The Synergy of India’s Universal Immunization Program and COVID-19 Vaccination Program. Vaccines [Internet]. 2023 Dec [cited 2025 Nov 6];11(12):1776. Available from: https://www.mdpi.com/2076-393X/11/12/1776

[55]	Arjun MC, Singh AK, Parida SP. CoWIN: The Future of Universal Immunization Program in India. Indian Journal of Community Medicine [Internet]. 2023 Aug [cited 2025 Nov 4];48(4):514. Available from: https://journals.lww.com/ijcm/fulltext/2023/48040/cowin__the_future_of_universal_immunization.3.aspx

[56]	Gurnani V, Singh P, Haldar P, Aggarwal MK, Agrahari K, Kashyap S, et al. Programmatic assessment of electronic Vaccine Intelligence Network (eVIN). PLOS ONE [Internet]. 2020 Nov 5 [cited 2025 Nov 4];15(11):e0241369. Available from: https://journals.plos.org/plosone/article?id=10.1371/journal.pone.0241369

[57]	Banerjee S, Banerjee S, Bhagi A, Sarkar A, Kapuria B, Desai S, et al. Blockchain technology for immunisation data storage in India: opportunities for population health innovation. BMJ Innovations [Internet]. 2022 Jan 1 [cited 2025 Nov 4];8(1). Available from: https://innovations.bmj.com/content/8/1/1

[58]	Agbo CC, Mahmoud QH, Eklund JM. Blockchain Technology in Healthcare: A Systematic Review. Healthcare [Internet]. 2019 June [cited 2025 Nov 4];7(2):56. Available from: https://www.mdpi.com/2227-9032/7/2/56

[59] Liu JC, Banerjee S, Bhagi A, Sarkar A, Kapuria B, Desai S, et al. Blockchain technology for immunisation documentation in India: findings from a simulation pilot. The Lancet Global Health [Internet]. 2021 Mar 1 [cited 2025 Nov 14];9:S22. Available from: https://www.sciencedirect.com/science/article/pii/S2214109X21001303

[60]	Chase R, Sanyal S, Singh P, Sharda S, Bhargava A, Raturi P, et al. Journey From a Digital Innovation to a Sustainable Health Worker Capacity-Building App in India: Experiences, Challenges, and Lessons Learned. Global Health: Science and Practice [Internet]. 2024 June 27 [cited 2025 Nov 4];12(3). Available from: https://www.ghspjournal.org/content/12/3/e2400006

[61] Dudeja N, Khan T, Varughese DT, Abraham SG, Ninan MM, Prasad CL, et al. Technologies for strengthening immunization coverage in India: a systematic review. The Lancet Regional Health - Southeast Asia [Internet]. 2024 Apr 1 [cited 2025 Nov 4];23:100251. Available from: https://www.sciencedirect.com/science/article/pii/S2772368223001117

[62]	Aggarwal S, Gupta P, Balaji S, Sharma S, Ghosh AK, Simmy, et al. Assessing the Feasibility of Drone‐Mediated Vaccine Delivery: An Exploratory Study. Health Sci Rep [Internet]. 2025 Jan 7 [cited 2025 Nov 4];8(1):e70208. Available from: https://pmc.ncbi.nlm.nih.gov/articles/PMC11705479/

[63]	Aggarwal S, Simmy S, Mahajan N, Nigam K. Challenges Experienced by Health Care Workers During Service Delivery in the Geographically Challenging Terrains of North-East India: Study Involving a Thematic Analysis. JMIR Formative Research [Internet]. 2024 Sept 10 [cited 2025 Nov 4];8(1):e57384. Available from: https://formative.jmir.org/2024/1/e57384

[64] Plewes K, Khonputsa P, Day NPJ, Lubell Y. Cost, health impacts and cost effectiveness of iceless refrigeration in India’s last-mile vaccine cold chain delivery. Transactions of The Royal Society of Tropical Medicine and Hygiene [Internet]. 2023 Apr 3 [cited 2025 Nov 4];117(4):310–2. Available from: https://academic.oup.com/trstmh/article/117/4/310/6947808

[65]	John Snow India. Immunization Technical Support Unit (ITSU) [Internet]. New Delhi: JSI India; [cited 2025 Nov 24]. Available from: https://jsiindia.in/our-projects/immunization-technical-support-unit-itsu/

[66]	Ministry of Health and Family Welfare (India). Minutes of Meeting – 18th NITAGI, July 2023 [Internet]. New Delhi: Ministry of Health and Family Welfare; 2023 [cited 2025 Nov 24]. Available from: /mnt/data/Minutes%20of%20Meeting-%2018TH%20NTAGI-%20July%20%272023.pdf

[67]	John J, Bavdekar A, Rongsen-Chandola T, Dutta S, Gupta M, Kanungo S, et al. Burden of typhoid and paratyphoid fever in India. N Engl J Med [Internet]. 2023 Apr 20 [cited 2025 Nov 4];388(16):1491–500. Available from: https://pmc.ncbi.nlm.nih.gov/articles/PMC10116367/

[68]	Typhoid Vaccines [Internet]. Take on Typhoid. [cited 2025 Nov 4]. Available from: https://www.coalitionagainsttyphoid.org/the-issues/typhoid-vaccines/

[69]	Date K, Shimpi R, Luby S, N R, Haldar P, Katkar A, et al. Decision Making and Implementation of the First Public Sector Introduction of Typhoid Conjugate Vaccine-Navi Mumbai, India, 2018. Clin Infect Dis. 2020 July 29;71(Suppl 2):S172–8.

[70]	Centre urges States to create awareness and take steps for prevention of cervical cancer among girl students [Internet]. [cited 2025 Nov 4]. Available from: https://www.pib.gov.in/www.pib.gov.in/Pressreleaseshare.aspx?PRID=1885597

[71]	Sharma H, Parekh S, Pujari P, Shewale S, Desai S, Bhatla N, et al. Immunogenicity and safety of a new quadrivalent HPV vaccine in girls and boys aged 9–14 years versus an established quadrivalent HPV vaccine in women aged 15–26 years in India: a randomised, active-controlled, multicentre, phase 2/3 trial. The Lancet Oncology [Internet]. 2023 Dec 1 [cited 2025 Nov 4];24(12):1321–33. Available from: https://www.thelancet.com/journals/lanonc/article/PIIS1470-2045(23)00480-1/abstract

[72]	Ghosh A, Chatterjee S, Dawn A, Das A. HPV Vaccines – An Overview. Indian J Dermatol [Internet]. 2025 [cited 2025 Nov 4];70(4):188–200. Available from: https://pmc.ncbi.nlm.nih.gov/articles/PMC12306887/

[73]	ICMR and Panacea Biotec initiate the First Dengue Vaccine Phase 3 Clinical Trial in India with Indigenous Dengue Vaccine, DengiAll [Internet]. [cited 2025 Nov 4]. Available from: https://www.pib.gov.in/www.pib.gov.in/Pressreleaseshare.aspx?PRID=2045090

[74]	Indian Council of Medical Research. EOI No ICMR-OIPM05: Malaria Vaccine ADFALCIVAX 2025 [Internet]. New Delhi: Indian Council of Medical Research; 2025 [cited 2025 Nov 24]. Available from: /mnt/data/1752741763_eoinoicmreoipm05malariavaccineadfalcivax2025.pdf

[75]	von Seidlein L. Roll out and prospects of the malaria vaccine R21/Matrix-M. PLoS Med [Internet]. 2025 Jan 17 [cited 2025 Nov 4];22(1):e1004515. Available from: https://pmc.ncbi.nlm.nih.gov/articles/PMC11741377/

[76]	Chitkara AJ, Parikh R, Mihalyi A, Kolhapure S. Hexavalent Vaccines in India: Current Status. Indian Pediatr [Internet]. 2019 Nov [cited 2025 Nov 4];56(11):939–50. Available from: http://link.springer.com/10.1007/s13312-019-1651-y
